# Suitability of three indicators measuring the quality of coordination within hospitals

**DOI:** 10.1186/1472-6963-10-93

**Published:** 2010-04-08

**Authors:** Etienne Minvielle, Henri Leleu, Frédéric Capuano, Catherine Grenier, Philippe Loirat, Laurent Degos

**Affiliations:** 1 Coordination for Measuring Performance Assuring Quality in Hospitals (COMPAQH), CERMES and Institut Gustave Roussy, INSERM (U988), 39, rue Camille Desmoulins 94805 Villejuif cedex, France; 2 Department of Quality, French Federation of Centre for Cancer (FNCLCC), 101, rue de Tolbiac - 75654 Paris Cedex 13, France; 3 High Authority for Health (HAS- Haute Autorité de Santé), 2, avenue du Stade de France, 93218 Saint-Denis La Plaine Cedex, France

## Abstract

**Background:**

Coordination within hospitals is a major attribute of medical care and influences quality of care. This study tested the validity of 3 indicators covering two key aspects of coordination: the transfer of written information between professionals (medical record content, radiology exam order) and the holding of multidisciplinary team meetings during treatment planning.

**Methods:**

The study was supervised by the French health authorities (COMPAQH project). Data for the three indicators were collected in a panel of 30 to 60 volunteer hospitals by 6 Clinical Research Assistants. The metrological qualities of the indicators were assessed: (i) Feasibility was assessed using a grid of 19 potential problems, (ii) Inter-observer reliability was given by the kappa coefficient () and internal consistency by Cronbach's alpha test, (iii) Discriminatory power was given by an analysis of inter-hospital variability using the Gini coefficient as a measure of dispersion.

**Results:**

Overall, 19281 data items were collected and analyzed. All three indicators presented acceptable feasibility and reliability (, 0.59 to 0.97) and showed wide differences among hospitals (Gini, 0.08 to 0.11), indicating that they are suitable for making comparisons among hospitals.

**Conclusion:**

This set of 3 indicators provides a proxy measurement of coordination. Further research on the indicators is needed to find out how they can generate a learning process. The medical record indicator has been included in the French national accreditation procedure for healthcare organisations. The two other indicators are currently being assessed for inclusion.

## Background

Patients are spending less time in hospital and are being managed by a greater number and diversity of health professionals. This means that better coordination of care is required. Donabedian described coordination of care as the "process by which the elements and relationships of medical care during any one sequence of care are fitted together in an overall design" [1]. This definition covers many aspects of coordination. Some of these relate to information technology, e.g. the development of the electronic medical record [2,3] and others to work organization, i.e. to pre-specified programs and mutual adjustment or feed-back [4-6]. Programming sets responsibilities and activities for a known and predictable task, and involves standardizing the information or the skills required. It tends to work well for routine procedures but not for highly uncertain procedures [7]. When circumstances or events cannot be foreseen, there is a need for feedback mechanisms such as process supervision or peer interaction [8].

Practically, this coordination of the work organization involves, for instance, scheduling, communicating, and responding to unexpected situations. Although these functions are sometimes forgotten by health professionals, they can impact on quality of care. An example of a key function is the transmission of written information on actions taken [9]. Another is setting up a longitudinal relationship with a single identifiable provider with optimal cooperation as goal [10].

The question thus arises how to assess through measurement, functions such as making available useful written information and cooperation among healthcare providers. Thus far, most coordination indicators have focused on primary care and the hospital-ambulatory sector interface [11,12] with coordination being linked to the notion of continuity of care as given by successive related sequences of medical care [13]. This article describes the development of three quality indicators (QIs) that measure either the availability of written information or staff cooperation in hospitals. The 3 QIs are the completeness and quality of the content of medical records, the completeness of the order for a radiology exam, and the holding of multidisciplinary team meetings (MDTM) on cancer patient management. The aim of the article is to describe the design of the QIs and discuss how they can be used to assess coordination within hospitals.

## Methods

The study was part of the COMPAQH project (COordination for Measuring Performance and Assuring Quality in Hospitals). The project is managed by INSERM (the French national Institute for Health and Medical Research) and is sponsored by the Ministry of Health and the *Haute Autorité de Santé *(HAS - National Authority for Health). Its objective is to develop QIs in order to monitor quality in French hospitals and to design ranking methods and pay-for-quality programs.

### QI Selection

In 2003, the French Ministry of Health and HAS listed 8 priority areas in need of quality improvement: "pain management", "practice guidelines", "organisational climate", "iatrogenic events", "nutritional disorders", "access to care", "taking account of patients' views", and "coordination of care". In 2006, a 9^th ^priority area was added to the list, namely, "continuity of care". COMPAQH has developed a total of 43 QIs relating to these 9 areas. Three of the 4 QIs for measuring" coordination of care" were selected for testing. The selection was based on an *ex ante *assessment of the frequency of lack of coordination, QI feasibility, and an upper limit of 3 QIs. The excluded QI was "operating room cancellations". The 3 selected QIs were:

QI 1: The completeness and quality of medical record content. Proper documentation of medical records helps in the sharing of useful information, reduces medical errors, and meets medical and legal requirements [14]. The quality of medical records is often poor in France. It is common to come across unsigned drug prescriptions and omission of a mention of the information a patient has been given [15].

QI 2: The completeness of the order for a radiology exam. Incomplete orders would be a major cause of low-quality image interpretation in hospitals [16]. According to the French Society of Radiology and the health authorities, clinicians and radiologists do not share all necessary information. This creates problems.

QI 3: The holding of multidisciplinary team meetings (MDTM) in the management of cancer patients. Since 2007, the treatment plan for each cancer patient must, by law, be discussed in a MDTM. It is assumed that a review of each case by staff with expertise in different fields enhances coordination of care.

A working group established a list of criteria and items for each QI on the basis of clinical practice guidelines, legal regulations, and consensus-based guidance. The working groups comprised 3 physicians and 2 nurses from different clinical specialties for QI 1, 5 radiologists for QI 2, and 5 representatives of different specialties (1 radiotherapist, 1 medical oncologist, 2 clinicians, and 1 nurse) for QI 3.

### QI development

#### Data collection

Participating hospitals were selected on the basis of type and location. Type was defined by size (number of beds) and status (public, private not-for-profit, private profit making). Each region of France was represented. Participation was voluntary. The number of hospitals ranged from 30 to 60 according to QI.

Data collection was in 6 steps: (1) Diffusion of an instructions brochure describing the data collection protocol and the items for each QI; (2) Nomination of a data collection manager in each hospital; (3) Random selection of medical records; (4) Data collection by 6 clinical research assistants (CRA) who completed the quality assessment grid for each selected medical record under the supervision of a physician; (5) Calculation of results; (6) Summary of the strengths and weaknesses of each QI, and diffusion of the validated instructions brochures to the bodies responsible for generalizing QI use (HAS accreditation procedure for health care organisations).

Indicator items are listed in Table 1. QI 1 (medical record quality) is given by a composite score based on 10 items for medical record content (item present or absent); QI 2 (quality of the order for the radiology exam) is given by a 5-item score; QI 3 (MDTM) is given by the compliance rate with the rule that a MDTM must be held for each cancer patient (mentioned in the patient file or not). Table 1 also gives the scoring system used, how the mean score was calculated, the number of random samples, and the number of acute care hospitals in which the QIs were assessed.

**Table 1:** QI description

	Patient record	Radiology exam order	MDTM
**Type of indicator**	Composite score of conformity criteria	Composite score of conformity criteria	Compliance rate
**Items (N)**	10 Presence of: - surgical report - obstetrics report - anesthestic record - transfusion record - outpatient prescription - admission documents - care and medical conclusions at admission - in-hospital during prescriptions - discharge report (incl. information on care delivered and conclusions reached) Overall organisation of record (incl. physician's name, patient's name, and date of admission)	4, Presence of:: - name of clinician ordering exam - type of exam requested - purpose of exam - patient information (name, age, key clinical history	1 Record of one MDTM with date and names of three professionals
**Scoring method**	1 (present)/0 (absent) for each item	1 (present for all 5 types of information)/0 (absence of one among the 5 type of information requested)	1 (present)/0 (absent) (record reviewed with written conclusion)
**Data source**	80 random records	130 random orders	60 random new oncology outpatient records
**Hospitals (N)**	36	22	22
**Calculations**	Mean score for each medical record. Mean score for all records in sample (with 99% and 90% confidence intervals). Overall mean score for all hospitals.	Mean score for all orders in sample (with 99% and 90% confidence intervals). Overall mean score for all hospitals.	Mean score for all records in sample (with 99% and 90% confidence intervals). Overall mean score for all hospitals.
**Observations (N)**	13 899	4004	1378

### QI testing

We determined QI feasibility, reproducibility, internal consistency, and discriminatory power. None of the QIs required adjustment. To assess feasibility, we used a validated grid of 19 items exploring 5 dimensions: acceptability by the institution and by health professionals, staff availability, understanding of indicator implementation, workload, and the IT system and organizational capacity to collect data [17]. The grid was completed by the 6 CRAs using 30 random records. We estimated reproducibility using kappa tests [18], internal consistency using Cronbach's alpha test, and discriminatory power using the Gini coefficient as a measure of dispersion in hospital scores. The Gini coefficient is a statistical measure commonly used in economics to assess differences in income or wealth. Discriminatory power is high if the Gini coefficient is under 0.2; variability is low if it is above 0.5 [19]. We used SAS version 8.1 (SAS Institute Inc, Cary, North Carolina) to perform the analyses.

## Results

### QI feasibility

The incidence of problems per item and per hospital was below 5% for medical record content and for compliance with holding a MDTM, but 14.5% for the radiology exam order (Table 2). The main problem with the radiology order QI was "not understood". This prompted rewording of the instructions for the QI by the working group. No CRA reported a problem that was an in-built limitation on the feasibility of this QI. No CRA or data collection manager reported a critical feasibility problem.

**Table 2:** Metrological qualities of each indicator

		Medical record	Radiology exam order	MDTM
**Feasibility**	% problems encountered	3.17	14.5	1
**Reliability**	Kappa score (range/item)	0.59 - 0.97	0.69 - 0.89	NA
**Internal consistency**	Cronbach's alpha	0.74	NA*	NA
**Discriminatory power**	Gini	0.08	0.17	0.11
	Std	11.1	21	15

NA: not applicable

NA as only one item considered

### QI testing

**Figure 1:**
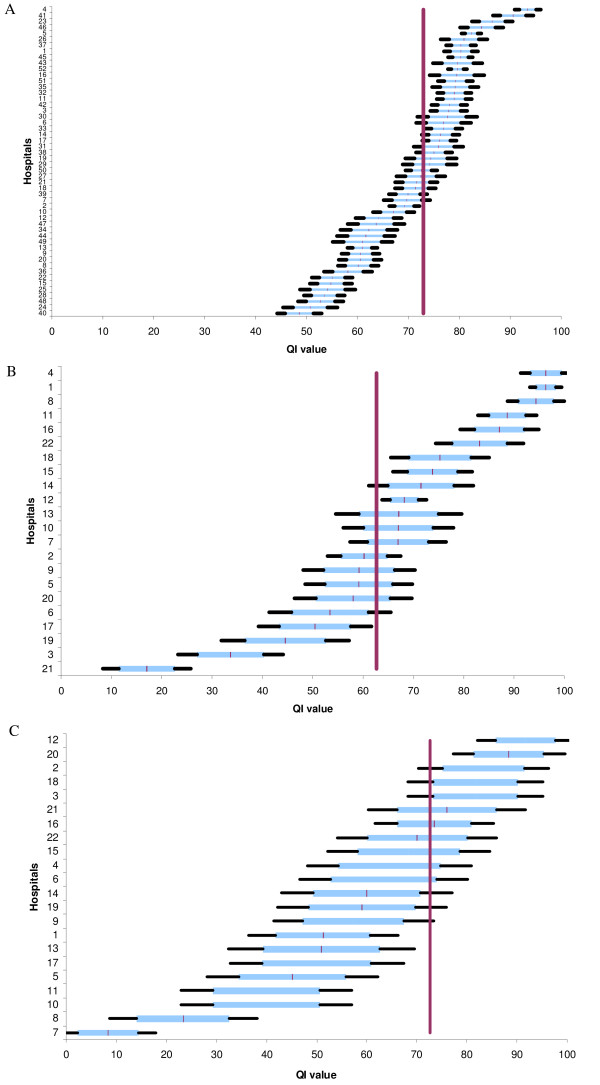
**Comparisons among hospitals using the indicator for (A) medical record content, (B) orders for radiology examinations, and (C) multidisciplinary team meetings**. The horizontal line shows the mean score (with 90 and 99% confidence intervals) for each hospital. The vertical line gives the overall mean score for all hospitals and is used for benchmarking. Hospitals are anonymously represented on the ordinate's axis.

The inter-observer reliability of the QIs for medical record content and the radiology exam order was satisfactory as shown by the kappa scores (Table 2). QI I had acceptable internal consistency (Cronbach coefficient: 0.74). The power of the QIs to discriminate among the hospitals was high despite the small sample size (Figure 1). The medical record score ranged from 39.6 ± 2% to 87.5 ± 2.5% according to hospital. The mean score was 72% for 36 hospitals. The radiology exam order score ranged from 16.9 ± 5.5% to 96.2 ± 3.1% with a mean score of 62.7% for a total of 22 hospitals. The MDTM score ranged from 8.3 ± 5.0% to 91.6 ± 6.0% with a mean score of 60.3% for 22 hospitals. The Gini coefficient was under 0.2 for all three QIs, which is an indication of high discriminatory power (Table 2).

## Discussion

We have developed 3 acceptable QIs covering two aspects of coordination (transfer of written information and adapting to the needs of others). But before concluding that they measure differences in coordination quality among hospitals, we need to consider several points.

First, the relationship between QI score and coordination quality may be subject to bias and erroneous interpretation. Selection bias may arise from incorrect sampling of medical records. To minimize such bias, we standardized the sampling method, gave confidence intervals to take sampling variability into account, used a standard grid, and checked for consistency during data collection. In the case of written information, differences in QI score between hospitals could have been due to different documentation processes rather than to genuine lack of information and/or cooperation between healthcare providers. However, whatever the cause of poor written information may be, it results in lack of coordination. In the case of MDTM, what was recorded may not have matched what actually happened. We can nevertheless reasonably assume that "false positives" (something recorded but not done) are far less frequent than "false negatives" (something done but not recorded) even if this has not been definitively established.

Second, coordination depends on many qualitative factors such as trust among staff members, experience in working together, the resilience of individuals and of the system, and the level of uncertainty encountered [20]. How meaningful are quantitative scores in such a context. Information can be transferred in ways that do not use written material as in the medical record or radiology exam order. Examples of information transfer are morning reports, verbal handovers, and informal conversations. All of these may be of high quality, but it is the written material that is somehow considered to provide the highest guarantee of coordination for understanding and sharing the meaning of previous actions.

We mentioned in the introduction part that coordination stresses the need to include both a standardized approach - programming - and a personal approach - feedback. Quantitative assessments like our set of 3 QIs seem to more cover aspects of standardization. Medical record content and the order for a radiology exam are standardized in order to create a common data core useful to all. We can say that feedback on cancer treatment plans is given to each person attending the MDTM. However, in this last case, true coordination really requires iterative feedback among health professionals as each decision is singular and its outcome difficult to predict. Iterative feedback should thus be captured by quality indicators and also by qualitative assessments.

The strength of the standards or guidelines underpinning QIs also needs to be considered. Guidelines are supported by an evidence base and, despite the wealth of literature, guidelines are not as common for evidence-based management as for evidence-based medicine. In the absence of strong evidence, QI use may not guarantee better coordination. We consider that there is a need to develop guidelines by applying conceptual frameworks to coordination, whilst at the same time complying with legal requirements for records and MDTMs.

A third point to be considered is that our 3 QIs explore aspects of coordination but not overall coordination within hospitals. It would be nice to know whether each hospital had similar results for all three QIs but, unfortunately, each QI was assessed in a different sample of hospitals. Nevertheless, wanting to tackle failures is already a step towards coming to grips with quality issues [21]. A QI can be used in a learning process to help understand the causes of failure, set improvement goals, and see whether the changes made have taught something [22,23]. To institute such a learning process based on results for QIs requires acceptance by health professionals. A QI may be rejected because it generates conflict. For instance, the order for a radiology exam is written by one set of professionals - clinicians - but evaluated by another set - radiologists. Such QIs may not be universally acceptable if there is a gap between their practical and theoretical use [24]. This is one reason why we worked in close collaboration with the healthcare professionals involved.

A final important point is that we need more evidence to be able to relate the quality of work coordination to outcome of care in hospitals [25,26].

## Conclusion

Our QIs are a first step towards measuring work coordination and resolving management failures. The QI for medical record content was included in 2008 in the national accreditation procedure for healthcare organizations (HCOs) run by HAS. Results for over 1300 HCOs reveal high variability among hospitals. The QIs for radiology orders and MDTMs are currently being assessed for inclusion in the accreditation procedure. Further research in management and social sciences is needed to ensure that our QIs are able (i) to capture a sufficient number of aspects of coordination, (ii) encourage a more in-depth analysis of work coordination by a wide range of health professionals, (iii) and help explain variability in clinical outcomes.

## Competing interests

The authors declare that they have no competing interests.

## Authors' contributions

EM has experience in quality indicator development and management. EM, HL, and contributed to writing the article. FC did the statistical analysis. PL, CG and LD critically revised the article. LD gave final approval of the submitted version. EM is guarantor.

## Pre-publication history

The pre-publication history for this paper can be accessed here:

http://www.biomedcentral.com/1472-6963/10/93/prepub
